# The impact of authors’ medical specialty on publication patterns and published results of adjuvant radiotherapy for WHO grade 2 meningiomas—a systematic review

**DOI:** 10.1007/s00701-021-04797-0

**Published:** 2021-03-29

**Authors:** Per Sveino Strand, Ole Solheim

**Affiliations:** 1 Department of Neuromedicine and Movement Science, Norwegian University of Science and Technology, Trondheim, Norway; 2 Department of Neurosurgery, St. Olavs Hospital, Trondheim University Hospital, Trondheim, Norway

**Keywords:** Atypical meningioma, Medical genealogy, Adjuvant radiotherapy, Brain tumor

## Abstract

**Background:**

The role of adjuvant radiotherapy after gross total resection (GTR) of WHO grade 2 meningioma remains unclear, and conflicting results have been published. We hypothesized that authors’ medical specialties could be associated with reported findings on the role of adjuvant radiotherapy after GTR of WHO grade 2 meningiomas.

**Method:**

A systematic review was conducted in Embase and Medline databases, in addition to screening of all relevant bibliographies. Articles including patients aged 18 years or older, with histologically confirmed WHO grade 2 meningioma, were included. We extracted data on medical subspecialties using the author list. We registered study design, median follow-up, number of included patients, WHO classification in use, and years of study inclusion.

**Results:**

Thirty-seven relevant studies were identified, where 34 (92%) were retrospective cohort studies, two studies (5%) were systematic reviews, and one study (3%) was a meta-analysis. If the last author was a radiation-oncologist, the study was more likely to favor adjuvant radiotherapy, and if a neurosurgeon was last author, the study was more likely to not advocate adjuvant radiotherapy (*p*=0.009). There was no significant association between study result and whether the study was published in a neurosurgical or oncological journal (*p*=0.802). There was no significant difference in follow-up time, years of inclusion, or number of included patients between studies favoring or not favoring adjuvant radiotherapy.

**Conclusions:**

In this systematic review of the literature, we found that if a radiation-oncologist was the last author of the study, the study was more likely to favor adjuvant radiotherapy after gross total resection of WHO grade 2 meningioma. Clinicians and researchers should be aware of a possible genealogy bias in the neuro-oncological literature.

## Background

The 2016 World Health Organization (WHO) Classification of Tumors the Central Nervous System stratifies meningiomas into three main groups: WHO grade 1 (benign), grade 2 (intermediate/atypical), and grade 3 (anaplastic/malignant) [17].

Regardless of WHO grade, primary management of meningiomas is maximal safe surgical resection, if treatment is necessary. In WHO grade 2 meningioma, there is a relative consensus that a subtotal resection (Simpson grade 4 and 5) is an insufficient treatment and adjuvant radiotherapy is usually administered [7]. However, the role of adjuvant radiotherapy after radiological gross total resection (Simpson grade 1–3) remains debated and unclear.

While some studies favor early adjuvant radiotherapy, arguing that it reduces the recurrence rate, overall survival, and progression free survival. [24, 27–29], other studies reach a different conclusion [8, 16, 19, 21]. Thus, management of atypical meningiomas currently varies across centers or caregivers [18].

Academic genealogy is the linking of scientists who have been academic mentors for each other and has been used to demonstrate the influence of mentors on students in several other fields [2, 22]. Recently, academic genealogy was used to review patterns in American publications on the survival effect of gross total surgical resection in patients with high-grade gliomas [11]. It was found that researchers belonging to different genealogies (e.g., neurosurgeons vs. radiation-oncologists) tend to reach contradictory findings and publish in different journals. This indicates that scientific echo chambers may develop and can be a source of bias in clinical outcome studies and in the assessment of the literature. We hypothesized that authors’ medical subspecialties could be associated with reported findings on the role of adjuvant radiotherapy after GTR of WHO grade 2 meningiomas and performed a systematic review of the literature to test the hypothesis.

## Methods

To identify all studies on adjuvant radiotherapy after GTR of atypical meningioma, we conducted a systematic search in Embase and PubMed, assisted by an experienced librarian. Two search term groups, one representing meningioma and one representing adjuvant radiotherapy, were combined with Boolean “and.” These search term groups contained both free text terms and controlled terms (MeSH in Pubmed, Emtree terms in Embase). Free-text terms applied were “adjuvant radiotherapy,” “radiotherapy,” “radiosurgery,” “atypical meningioma,” “who grade two meningioma,” and “who grade II meningioma.” MeSH and Emtree terms applied were “Radiosurgery,” “Radiotherapy,” or “Radiotherapy, adjuvant,” and “Meningioma.”

**Fig. 1:**
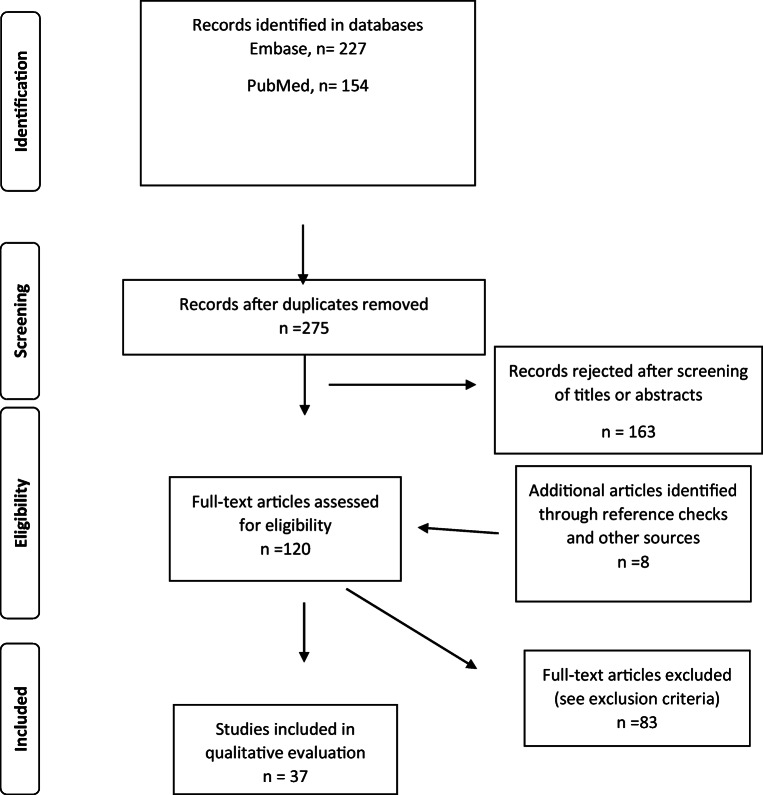
Flowchart for inclusion of articles

A flow-chart of the inclusion process is presented in Fig. 1. We included only human studies that compared adjuvant radiotherapy vs. no adjuvant radiotherapy after a GTR for atypical meningioma in ≥10 patients ≥ 18 years. Only articles published in English were included. Due to advances in both pre-and perioperative imaging, radiotherapy, and surgical techniques, articles published before 1990 were excluded. Case-reports and conference abstracts were excluded, as well as mixed WHO grade 2/3 series where separate data for WHO grade 2 were not presented. The bibliography of the included articles was screened for relevant studies. Studies from meta-analyses which met our inclusion criteria were also included.

We extracted data on medical subspecialties using the author list. In cases of doubt, we sent an email to the author(s). The author lists were reviewed to see whether there was a neurosurgeon in the study group. We identified one first author without a medical degree (Bachelor of Science), and one last author who was a pathologist. These authors were classified as “other.” Furthermore, we registered median follow-up time, the number of included patients, and years of study inclusion. In studies with different follow-up time, the longest follow-up was reported. In mixed populations, only results in WHO-2 patient numbers were reviewed and reported.

## Statistics

Statistical analyses were performed with IBS SPSS Statistics version 25.0 (IBM, Armonk, New York). Kolmogorov-Smirnov test and Q-Q-plots were used to determine normal distribution of data. Differences between groups were assessed using one-way analysis of variance and Fisher’s exact test, for continuous and categorical variables, respectively. The Kruskal-Wallis test was used for skewed data. Statistical significance level was set to *p* < 0.05.

## Results

Study characteristics are presented in Table 1. After removal of duplicates, screening on titles, abstracts, and full-text analysis, we identified 29 studies that met our inclusion criteria. Screening the bibliography of the included studies, we identified eight additional articles, resulting in inclusion of 37 eligible studies. Thirty-four studies (92%) were retrospective cohort-studies, two (5%) were systematic reviews, and one study (3%) was a meta-analysis. Notably, we could not identify any prospective studies, controlled studies, or randomized-controlled trials. Most of the studies (76%) used multiple endpoints. Eleven studies (30%) concluded in favor of adjuvant radiotherapy after GTR, 21 studies (57%) did not favor adjuvant radiotherapy, while five studies (14%) reported inconclusive results.

**Table 1:** Study characteristics

Study-design	
Retrospective cohort-study Systematic review Meta-analysis	34 (91.9%) 2 (5.4%) 1 (2.7%)
Endpoint(s)	
Overall survival Progression-free survival Local recurrence (y/n) Multiple endpoints	2 (5.4%) 2 (5.4%) 5 (13.5%) 28 (75.7%)
Multi-center study	
Yes No	9 (23.7%) 28 (75.7%)
Registry-based study	
Yes No	3 (8.1%) 34 (91.9%)
WHO classification	
1993 2000 2007 2016 Multiple grading systems Not reported	1 (2.7%) 4 (10.8%) 14 (37.8%) 2 (5.4%) 9 (24.3%) 7 (18.9%)
Tumor histology included	
Atypical meningioma exclusively Multiple tumor entities	28 (75.7%) 9 (24.3%)
Study conclusions	
In favor of radiotherapy Not in favor of radiotherapy Inconclusive	11 (29.7%) 21 (56.8%) 5 (13.5%)

As seen in Table 2, if the last author was a radiation-oncologist, the study was more likely to favor adjuvant radiotherapy 63.6 vs. 9.5% (*p*=0.009). Furthermore, studies in favor of radiotherapy tended to be smaller than studies not favoring radiotherapy, although this difference did not reach significance (*p*=0.070). There was no statistically significant difference in first authorships, type of journal, presence of a neurosurgeon in the research group, duration of follow-up, or years of inclusion across studies with the different conclusions.

**Table 2:** In favor or not in favor of radiotherapy

	In favor of radiotherapy *N*=11	Not in favor of radiotherapy *N*=21	Inconclusive *N*=5	*p* value
First author				
Neurosurgeon	4 (36.4%)	15 (71.4%)	2 (40.0%)	0.134
Radiation-oncologist	7 (63.6%)	5 (23.8%)	3 (60.0%)	
Other	0 (0.0%)	1 (4.8%)	0 (0.0%)	
Last author				
Neurosurgeon	4 (36.4%)	18 (85.7%)	4 (80%)	0.009
Radiation-oncologist	7 (63.6%)	2 (9.5%)	1 (1%)	
Other	0 (0.0%)	1 (4.8%)	0 (0.0%)	
Journal				
Neurosurgical	5 (45.4%)	13 (61.9%)	3 (60.0%)	0.802
Oncological	6 (54.5%)	7 (33.3%)	2 (40.0%)	
Other	0 (0.0%)	1 (4.8%)	0 (0.0%)	
Neurosurgeon in the research group				
Yes	9 (81.8%)	20 (95.2%)	0 (0%)	0.532
No	2 (18.2%)	1 (4.8%)	5 (100%)	
Number of patients (median, IQR)	64.0 (45–155)	133 (87–186)	40 (16–407)	0.070
Median follow-up in months (mean, SD)	53.6 (±12.3)	55.1 (±19.3)	43.9 (±17.2)	0.510
Years of inclusion (median, IQR)	12.0 (9–15)	12.0 (9–14.5)	15.0 (12–28)	0.502

A separate analysis was done for primary studies only. The results were similar, except for that number of patients included reached statistical significance (median 64, vs. 123 vs. 22 in the studies favoring radiotherapy, not favoring radiotherapy, and inconclusive studies, respectively, *p*=0.010).

## Discussion

In this systematic review of the literature, we found that if a radiation-oncologist was the last author, the study was more likely to conclude in favor of adjuvant radiotherapy after gross-total tumor resection of WHO grade 2 meningioma. This may be an indication of bias in the literature. The reasons may be complex, ranging from assessment bias (e.g., definition of progression), to publication bias and confirmation bias when clinicians review their own practice. This should be kept in mind when reading, reviewing, or conducting meta-analyses of the literature in many fields of medicine, especially when relying on low-level evidence. The present review study adds to the recent work addressing how medical genealogy may affect study results and publication patterns [11–13]. The authors of these studies have introduced the term “genealogy bias.”

The topic of adjuvant radiotherapy for atypical meningioma after GTR remains controversial. The European Association of Neuro-oncology (EANO) emphasize that the role of adjuvant radiotherapy after GTR is unclear [7]. As seen in our systematic review, available studies are of rather low quality; most studies are retrospective cohorts, prone to several forms of bias. In addition, three out of four studies had multiple endpoints, and the median duration of follow-up was rather short for many studies, and it is therefore possible that some studies miss late tumor recurrences. In incurable cases, giving all treatment options up-front may perhaps increase time to recurrence, but it will also make treatment options fewer at recurrence. In meningioma, both survival studies and studies reporting patient-reported outcomes are still seldom in the published literature.

A meta-analysis that included 757 patients reported improved 5-year local control rates and decreased recurrence rates for patients that received adjuvant radiotherapy [10]. However, they found no significant differences in overall survival. Yet, the meta-analysis is exclusively made up of retrospective and non-randomized data. We sat a publication cut-off for 1990 in our inclusion criteria, yet some studies included patients back to the 1960s [10], and during this time period, major improvements have been seen in diagnostic imaging, surgical techniques, and adjuvant radiotherapy.

The histological heterogeneity of meningiomas was first recognized by Cushing [6], and meningioma has been subject to several histological classifications and re-classifications over the years. Depending on the classification used, institution tradition, and a lag-time to implementation [15], the incidence of atypical meningioma range from 5 to 35% [4]. The later reclassifications of meningiomas have led to a substantial increase in the prevalence of atypical meningioma [20, 26]. Thus, the relevance of older studies on the subject can be questionable. Some studies included atypical meningiomas diagnosed using the 1993 WHO system, which may result in different responses to radiotherapy compared to tumors diagnosed using the 2000 or 2007 WHO systems [1, 5]. Moreover, although it is generally accepted that a GTR is defined as Simpson grade 1–3 [3, 23], one study defined GTR as Simpson grade 1 [9]. Furthermore, there are variations in both dose and timing of RT.

More prospective data will be available from the ongoing randomized-controlled study “Radiation versus Observation following surgical resection of Atypical Meningioma” (ROAM, EORTC 1308) [14]. The study opened in 2015 and aims to randomize 190 patients after Simpson grade 1–3 resection to either early radiotherapy or observation and will hopefully shed light on the controversy of adjuvant radiotherapy vs. a strategy of active monitoring, at least for progression free survival. Unfortunately, the ROAM trial has had problems with recruitment. A recent qualitative study addresses challenges that clinicians face when communicating that there is no good basis for a choice between two or more treatments to patients. Interestingly, the study reported that neurosurgeons not involved in the trial advised patients not to participate in the trial [25]. Another study has demonstrated a significant difference of opinion about the role of adjuvant radiotherapy after resection of WHO grade 2 meningiomas in different centers in the UK and the Republic of Ireland [18]. However, reasons for this clinical discrepancy have not been addressed. Could it be that our own medical specialties and scientific herds have a greater impact on our practice of medicine more than we would like to acknowledge?

The findings from the present review indicate that there is an association between last authorship and study conclusion on the controversial matter of adjuvant radiotherapy after GTR of WHO grade 2 meningiomas. Traditionally, the last author is a leader of the research group, but not necessarily. The use of first and last authorships as a marker of genealogy may therefore be questioned. Although not statistically different, also the majority of papers published with a radiation-oncologist as first authors conclude in favor of adjuvant radiotherapy while the majority of papers published with a neurosurgeon as first author conclude against adjuvant radiotherapy.

## Conclusion

In this systematic review of the literature, we found that if a radiation-oncologist was the last author of the study, the study was more likely to favor adjuvant radiotherapy after gross total resection of WHO grade 2 meningioma. Clinicians and researchers should be aware of a possible genealogy bias in the neuro-oncological literature.
